# NSAIDs disrupt intestinal homeostasis by suppressing macroautophagy in intestinal epithelial cells

**DOI:** 10.1038/s41598-019-51067-2

**Published:** 2019-10-10

**Authors:** Ana M. Chamoun-Emanuelli, Laura K. Bryan, Noah D. Cohen, Taylor L. Tetrault, Joseph A. Szule, Rola Barhoumi, Canaan M. Whitfield-Cargile

**Affiliations:** 1 Department of Large Animal Clinical Sciences, College of Veterinary Medicine & Biomedical Sciences, Texas A&M University, College Station, Texas, United States of America; 2 Department of Veterinary Pathobiology, College of Veterinary Medicine & Biomedical Sciences, Texas A&M University, College Station, Texas, United States of America; 3 Department of Veterinary Integrative Biosciences, College of Veterinary Medicine & Biomedical Sciences, Texas A&M University, College Station, Texas, United States of America

**Keywords:** Gastrointestinal diseases, Small intestine

## Abstract

Small intestinal damage induced by nonsteroidal anti-inflammatory drugs (NSAIDs) remains an under-recognized clinical disorder. The incomplete understanding of the pathophysiology has hampered the development of prevention and treatment strategies leading to the high morbidity and mortality rates. NSAIDs are known to modulate macroautophagy, a process indispensable for intestinal homeostasis. Whether NSAIDs stimulate or repress macroautophagy and how this correlates with the clinical manifestations of NSAID enteropathy, however, remains unknown. The objectives of this study were to determine whether NSAIDs impaired macroautophagy and how this affects macroautophagy-regulated intestinal epithelial cell (IEC) processes essential for intestinal homeostasis (*i*.*e*., clearance of invading pathogens, secretion and composition of mucus building blocks, and inflammatory response). We show that NSAID treatment of IECs inhibits macroautophagy *in vitro* and *in vivo*. This inhibition was likely attributed to a reduction in the area and/or distribution of lysosomes available for degradation of macroautophagy-targeted cargo. Importantly, IEC regulatory processes necessary for intestinal homeostasis and dependent on macroautophagy were dysfunctional in the presence of NSAIDs. Since macroautophagy is essential for gastrointestinal health, NSAID-induced inhibition of macroautophagy might contribute to the severity of intestinal injury by compromising the integrity of the mucosal barrier, preventing the clearance of invading microbes, and exacerbating the inflammatory response.

## Introduction

Nonsteroidal anti-inflammatory drugs (NSAIDs) are among the most frequently used class of medications worldwide^1^. Despite their effective anti-inflammatory and analgesic properties, the use of NSAIDs is associated with adverse effects to the upper (stomach and duodenum) and lower (jejunum and ileum, referred to as enteropathy) gastrointestinal (GI) tract. NSAID-induced gastropathy was first recognized in 1938^2^. The accessibility of the upper GI tract via gastroscopy, and the well-characterized pathogenesis of NSAID gastropathy have resulted in the development of effective treatment and management options^3^. Conversely, NSAID enteropathy remains an under-recognized clinical disorder with no effective therapeutic or preventative strategy(ies), largely because diagnosis is more complex and the pathophysiology is not completely understood^4^. In people, NSAID enteropathy is characterized by erosion and ulceration of the distal small intestine that can lead to life-threating complications including intra-abdominal hemorrhage, intestinal perforation, and intestinal strictures^5^. The mechanism of NSAID enteropathy remains ill-defined and is likely multifactorial, with topical effects, deficiency of prostaglandins, NSAID-microbiota interactions, microbial dysbiosis, mitochondrial dysfunction, increased intestinal permeability and motility, and generation of reactive oxidative species identified as contributing factors^4^. Development of therapeutic approaches guided by these factors, however, have not reduced the annual burden of NSAID enteropathy, underscoring the gap in mechanistic understanding^6^. Importantly, NSAID enteropathy is estimated to account for 100,000 hospitalizations and 16,500 deaths annually in the U.S. alone^7^, although, these numbers likely underestimate the burden of this disease^8^. In addition, approximately 30 million people consume NSAIDs every day, of which 2/3 of short- and long-term users develop some degree of small intestinal damage^8^, highlighting the need for an effective preventive and/or therapeutic strategy(ies).

Intestinal homeostasis requires the cooperative action of microbial cells (*i*.*e*., the microbiota), epithelial cells, and immune cells to promote the digestion and absorption of nutrients while maintaining a physiochemical barrier between luminal contents and the lamina propria of the intestinal wall. Despite the symbiotic relationship, the rich number of microorganisms in the GI tract pose a continuous threat to cause tissue damage and inflammation. Thus, intestinal epithelial cells (IECs) have evolved numerous strategies to prevent harmful reactions, including: (i) the secretion of mucin and antimicrobial peptides that shape the composition and location of the microbiota; (ii) suppressing the survival and dissemination of microorganisms that invade the mucosal layer; and, (iii) coordinating the responses of sub-epithelial immune cells^9^. All of these mechanisms are partly regulated by macroautophagy and its inhibition can negatively impact IEC function leading to microbial dysbiosis, invasion and dissemination of opportunistic pathogens, uncontrolled inflammatory responses and cell death^9^. Macroautophagy, hereon designated as autophagy, is a catabolic process where unneeded or damaged organelles or cytosolic components are targeted for lysosomal degradation^10^. Perturbations in autophagy have been linked to GI-associated diseases and disorders, especially in the context of inflammatory bowel disease (IBD), where a subset of patients have defects in autophagy-related genes^11^. Interestingly, NSAIDs have been shown to modulate autophagy in IECs, albeit with conflicting results, and patients with IBD are advised to refrain from NSAID intake as this may increase the risk of IBD onset and/or relapse^12^. Specifically, treatment of rat enterocytes with the NSAID indomethacin initially induced (6 h) lipophagy as a cytoprotective mechanism in a response to endoplasmic reticulum stress and lipid droplet (LD) accumulation. After 24 hours, however, indomethacin treatment reduced expression of lysosomal associated membrane protein 2 (LAMP-2). Since this protein is required for fusion of lysosomes and autophagosome vesicles, indomethacin treated cells displayed inhibition of autophagic flux leading to LD accumulation and lipoapoptosis^13^. Three years later, Harada *et al*., demonstrated that indomethacin treatment of IECs uncontrollably activates autophagy leading to cell death and small intestinal injury in rats^14^. While both studies demonstrate the effects of indomethacin on autophagy, one suggests that indomethacin leads to uncontrolled autophagy activation and cell death, while the other suggests that initially indomethacin induces autophagy but ultimately results in inhibition of autophagic flux prompting cell apoptosis. Given the importance of autophagy on intestinal homeostasis and the contradictory results of NSAIDs effects on autophagy, the objectives of this study were to i) characterize the effects of NSAIDs on autophagy in intestinal epithelial cells both *in vitro* and *in vivo* and ii) determine its impact on autophagy-dependent IEC functions indispensable for intestinal homeostasis. We show that NSAIDs inhibited autophagic flux *in vitro*. This inhibition was attributed to a reduction in the area and/or distribution of lysosomal vesicles in NSAID-treated cells. A similar pattern was observed *in vivo*, with indomethacin-treated mice displaying impaired autophagic flux accompanied by a reduction in lysosome staining. Importantly, IEC functions essential for intestinal homeostasis and partly regulated by autophagy, were dysfunctional in the presence of NSAIDs both *in vitro* and *in vivo*, suggesting that NSAIDs disrupt intestinal homeostasis as a result of autophagy inhibition.

## Results

### NSAIDs inhibit autophagic flux in IECs

**Figure 1:**
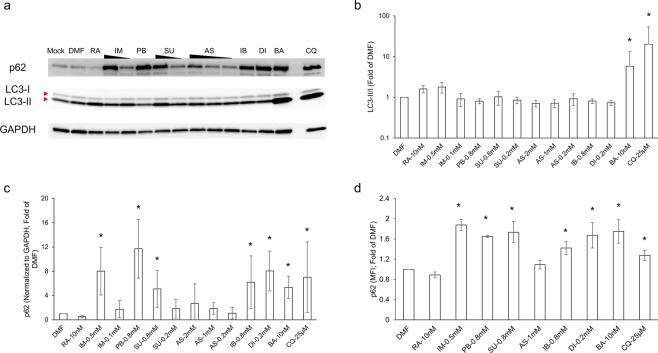
NSAIDs inhibit autophagic flux in IECs. YAMC cells were exposed to different classes of NSAIDs. Twenty-four hours later, autophagy markers LC3 and p62 were analyzed via western blot and/or flow cytometry. Full-length blots are presented in Supplementary Fig. 5. Values and error bars represent the average and 95% confidence intervals, respectively, of at least two independent experiments. DMF: Dimethylformamide; RA: rapamycin; IM: indomethacin; PB: phenylbutazone, SU: sulindac; AS: aspirin; IB: ibuprofen; DI: diclofenac; BA: bafilomycin; TFP: trifluoperazine; CQ: chloroquine; *p < 0.05, N.S. = no statistical significance.

NSAIDs have been shown to modulate the autophagy process. Most of the work to date, however, has focused on their ability to induce autophagy, with only two reports suggesting that NSAIDs can also inhibit completion of the autophagy process (*i*.*e*., autophagic flux)^13,15^. Moreover, the majority of studies have used either cancer-derived cell lines, where the autophagy process may be altered to benefit cell survival and cancer progression, or cells derived from newborn rodents that only retained some of the properties of the GI mucosa^16^. The current study takes advantage of a conditionally immortalized young adult mouse colonic (YAMC) epithelial cell line. These cells express a heat-labile polyomavirus simian virus 40 (SV40) large T-antigen under the control of an interferon (IFN)-γ inducible promoter. At 33 **°**C and in the presence of IFN-γ, the SV40 large T-antigen binds to and inactivates p53, thus driving cell proliferation. However, under non-permissive conditions, the temperature sensitive SV40 large T-antigen is inactive causing the cells to behave as normal, non-proliferating colonic epithelial cells^17^, thereby providing a more realistic view on the impact of autophagy on “naïve” cells. Autophagy is a coordinated multi-step process involving the formation and elongation of double membrane vesicles, known as autophagosomes, where sequestered cargo is delivered to lysosomes for degradation. After its induction, microtubule-associated protein 1A/1B light chain-3 (LC3-I) is covalently linked to lipid phosphatidylethanolamine in the nascent autophagosome where, in conjunction with sequestosome-1 protein (p62), cargo is engulfed and delivered to lysosomes for degradation^10^. Conversion of LC3-I into LC3-II through lipidation is routinely used as an indicator of autophagy induction, as it is required for autophagosome formation. However, since a portion of LC3-II is subject to degradation in the autolysosome, its accumulation may also indicate inhibition of autophagic flux. Conversely, autophagosome sequestered p62 along with engulfed cargo is degraded in the autolysosome and thus has been used as a marker of autophagy completion (autophagic flux)^18^. To assess the effect of NSAIDs on autophagy, both markers were evaluated thereby providing a more reliable measure of bona fide autophagy^18^. As controls, rapamycin, bafilomycin and chloroquine were included. Rapamycin induces autophagy via mammalian Target Of Rapamycin (mTOR) inhibition, promoting LC3-I lipidation and degradation of autophagosome-sequestered cargo^19^. In contrast, chloroquine, is a weak base that penetrates lysosomes and increase their intracellular pH, whereas bafilomycin, is an inhibitor of vacuolar-type H+ -ATPase and prevents endosome/lysosome acidification thereby inhibiting autophagic flux^20–22^. As shown in Fig. 1, all NSAIDs, independent of their classification, were able to inhibit autophagic flux as gauged by the significant p62 accumulation (Fig. 1c,d), albeit at different levels compared to vehicle control dimethylformamide (DMF). The LC3-II/I ratio was not significantly altered by NSAIDs *in vitro* (Fig. 1b). Autophagy inducer, rapamycin, displayed reduced levels of p62 relative to DMF, indicative of degradation of encapsulated material. As expected, treatment of YAMC cells with lysosomal acidification inhibitors, bafilomycin and chloroquine, revealed both an increase in LC3-II/I ratio and p62 accumulation, suggesting that autophagosomes formed are incapable of cargo degradation.

**Figure 2:**
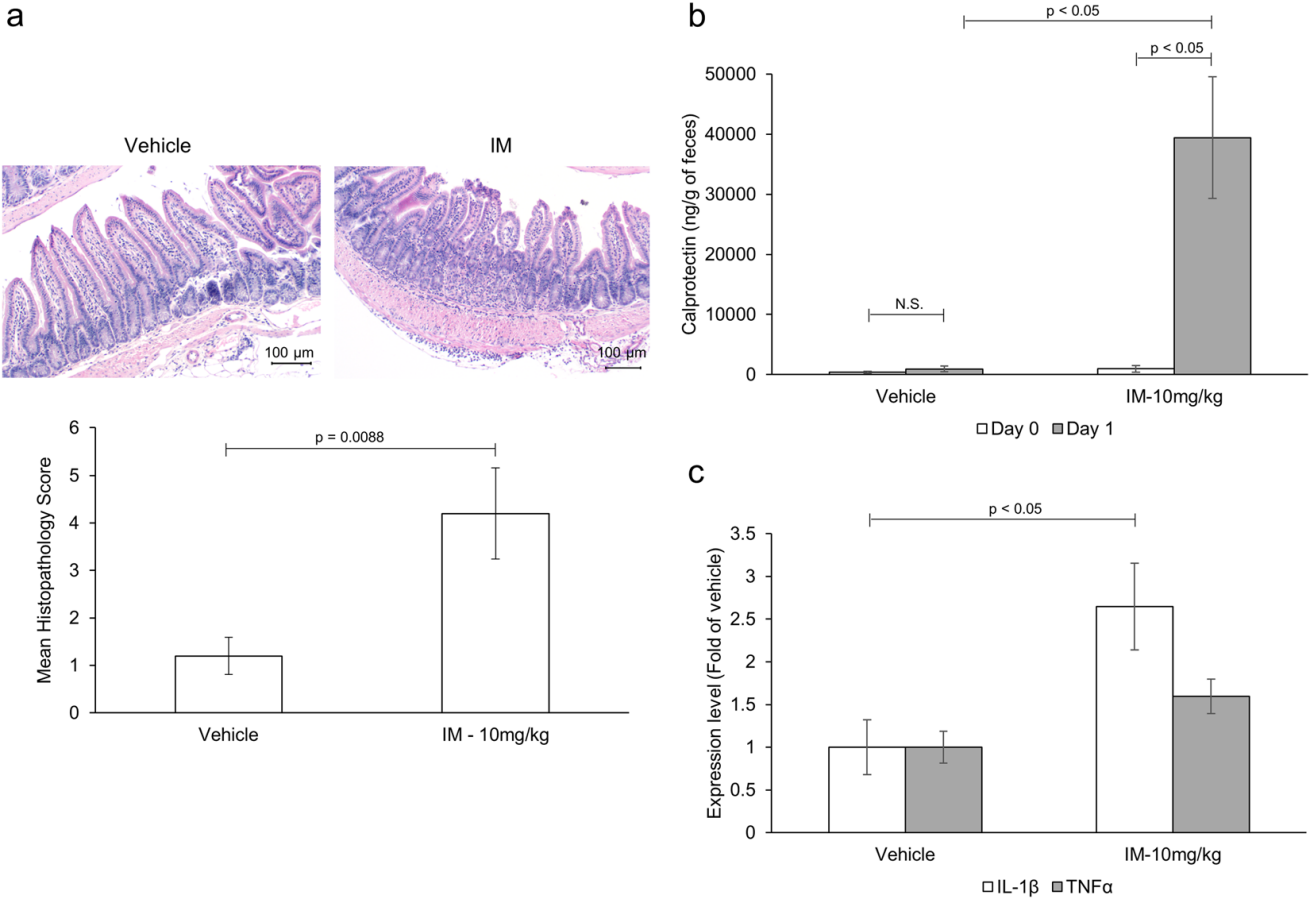
Indomethacin induces small intestinal injury. Mice (n = 5/group) were administered indomethacin (10 mg/kg) or vehicle control. Twenty-four hours later, the inflammatory response was evaluated via (**a**) microscopic pathology of the small intestine (**b**) increase in fecal calprotectin levels and (**c**) mRNA expression of pro-inflammatory cytokines in small intestinal tissue. Values and error bars represent the average and 95% confidence intervals, respectively.

**Figure 3:**
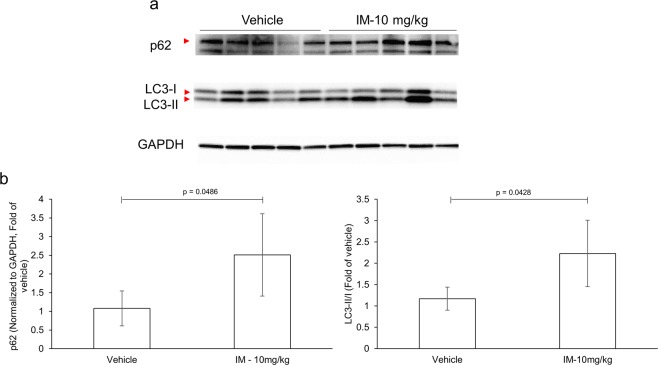
Indomethacin inhibits autophagic flux *in vivo*. Mice (n = 5/group) were administered indomethacin (10 mg/kg) or vehicle control. Twenty-four hours later, the levels of autophagy markers, LC3 and p62, in small intestinal tissue were analyzed via western blot (**a**). The different lanes represent the different animals used per group. Bands corresponding to the proteins of interest are indicated by the red arrow head. (**b**) Quantitative densitometry of blots in panel a show autophagy substrate p62 in small intestinal tissue from indomethacin- and control-treated mice (left panel). Relative ratio of LC3-II/I in small intestinal tissue of indomethacin- and control-treated mice (right panel). Full-length blots are presented in Supplementary Fig. 6. Values and error bars represent the average and 95% confidence intervals, respectively.

To evaluate the ability of NSAIDs to recapitulate inhibition of autophagy *in vivo*, mice (n = 3 to 5/group) were administered a single dose of the NSAID indomethacin (10 mg/kg) or vehicle control. Twenty-four hours later, mice were euthanized and samples were harvested. Indomethacin-treated mice demonstrated small intestinal damage characteristic of NSAID enteropathy as confirmed by microscopic pathology scores (Fig. 2a)^23^. In addition fecal calprotectin, a marker of neutrophil infiltration routinely used for detection and monitoring of gastrointestinal inflammation, including NSAID enteropathy, (Fig. 2b), and gene expression of pro-inflammatory cytokines (Fig. 2c) were elevated in indomethacin-treated animals compared to control mice^24,25^. Intestinal mucosal lysates collected from indomethacin-treated mice displayed p62 accumulation and an overall increase in LC3-II/I ratio compared to control animals indicating that, analogous to our *in vitro* studies, indomethacin inhibits autophagic flux *in vivo* (Fig. 3a,b).

### NSAIDs reduce the area of lysosomal vesicles in IECs

**Figure 4:**
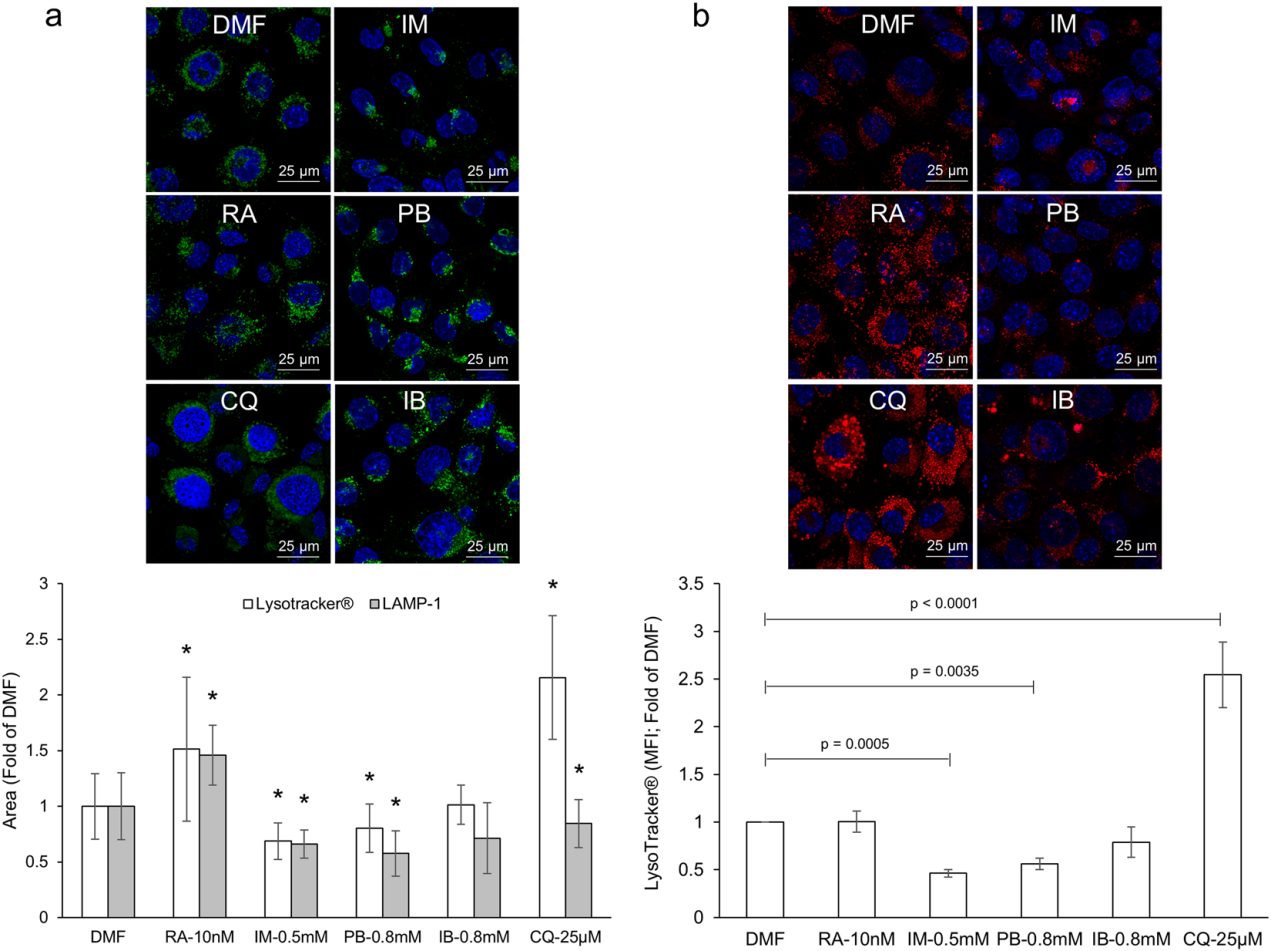
NSAIDs reduce the area of lysosomal vesicles *in vitro*. YAMC cells were exposed to different classes of NSAIDs for 24 h. The area and subcellular distribution of lysosomal vesicles was assessed via microscopy and flow cytometry. (**a**) Representative images (upper panel) and quantitative analysis of cells stained for LAMP-1 (lower panel) 24 h post drug treatment. (**b**) Representative images (upper panel) and quantitative analysis of cells stained with Lysotracker® probe (lower panel) 24 h post drug treatment. Values and error bars represent the average and 95% confidence intervals, respectively. DMF: Dimethylformamide; IM: indomethacin; PB: phenylbutazone, IB: ibuprofen; RA: rapamycin; CQ: chloroquine.

**Figure 5:**
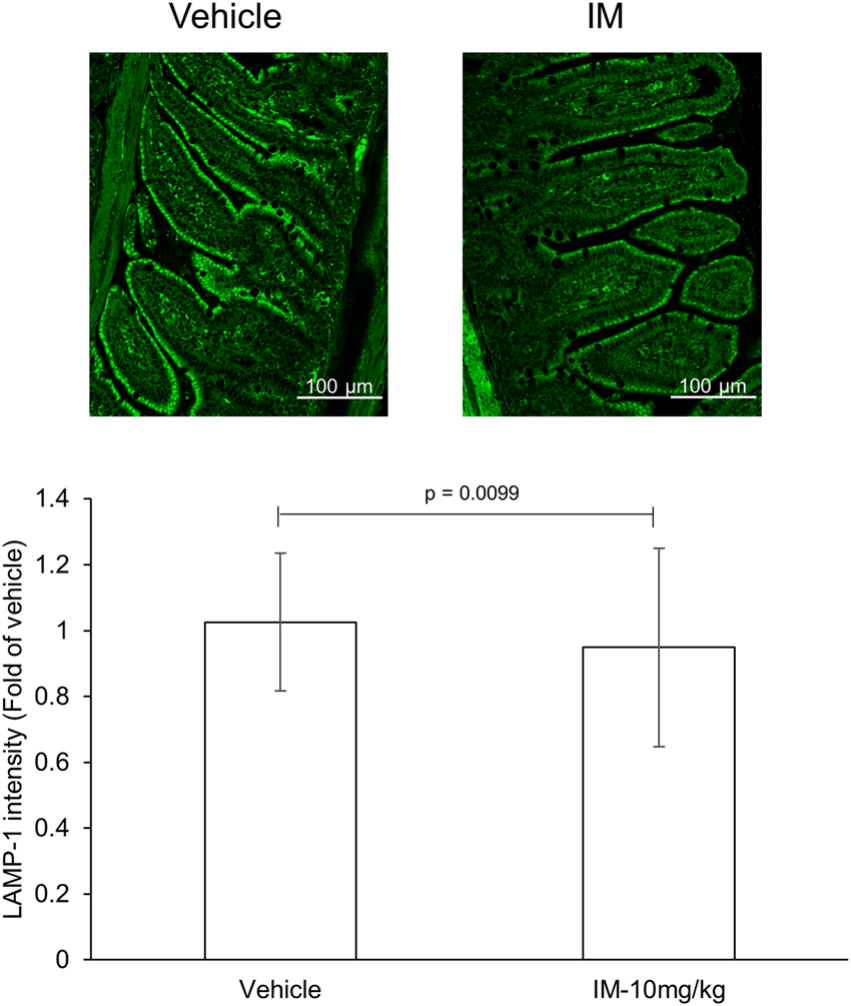
NSAIDs reduce the number of lysosomal vesicles *in vivo*. Mice (n = 4/group) were administered a single dose of indomethacin (10 mg/kg) or vehicle control. Twenty-four hours later, quantification of lysosomal vesicles was evaluated via microscopy. Representative images (upper panel) and relative staining intensity of LAMP-1 (lower panel) from small intestinal sections of indomethacin- and control-treated mice. Values and error bars represent the average and 95% confidence intervals, respectively.

The simultaneous accumulation of LC3-II and p62 in NSAID-treated mice and YAMC cells (Figs 1 and 3) suggests that autophagy is perturbed at a stage following induction. This pattern suggests that induction of autophagy is not altered, prompting us to evaluate the effect of NSAIDs on lysosomal vesicles, a required organelle for the final stages of autophagy (lysosome-autophagosome fusion). Briefly, YAMC cells were exposed to NSAIDs, autophagy modulators, or vehicle control for 24 h. The subcellular distribution of lysosomes in these cells was assessed via lysosomal associated membrane protein 1 (LAMP-1) staining. Indomethacin, phenylbutazone, and ibuprofen were selected as representatives of the different NSAID classes. YAMC cells treated with these NSAIDs displayed an altered distribution of lysosomes, albeit at different levels (Fig. 4a; upper panel). Clustering of these vesicles near or at the perinuclear region was evident as quantified by the total area of lysosomes per field. (Fig. 4a; lower panel). The positive controls rapamycin and chloroquine did not affect the distribution of lysosomes compared to DMF control. To ensure that LAMP-1 staining in NSAID-treated cells was representative of lysosomal vesicles, and not of the ability of NSAIDs to hinder trafficking of LAMP-1 between the Golgi complex and the nascent lysosome, vesicles were stained with pH-sensitive probe, LysoTracker®. Similar to LAMP-1, NSAID-treated cells displayed clustering of LysoTracker® positive vesicles near or at the perinuclear region and a reduction in the area and intensity of LysoTracker® positive vesicles as quantified via microscopy and flow cytometry (Fig. 4a; lower panel and 4b). Positive control rapamycin did not affect the subcellular distribution of lysosomes while significantly increasing the area of LAMP-1/Lysotracker ® positive vesicles. The lysosomotropic agent, chloroquine, displayed a similar lysosome distribution pattern as DMF control; however, an increase in the size of LysoTracker® positive vesicles was apparent. This phenomena is not exclusive to YAMC cells and has been attributed to an osmotic imbalance, whereby disruption of ion homeostasis by lysosomotropic cationic drugs results in water influx and swelling of lysosomal vesicles^20,26,27^. Furthermore, quantification of LAMP-1 from small intestinal tissues of indomethacin-treated mice resulted in a reduced LAMP-1 intensity compared to vehicle control, implying a reduction in the number of lysosomal vesicles (Fig. 5). Collectively, these results suggest that NSAID-induced autophagy inhibition may be explained by a reduction in the area and/or distribution of lysosomal vesicles, organelles required for the degradation of autophagosome-encapsulated material.

### Indomethacin disrupts intestinal homeostasis

**Figure 6:**
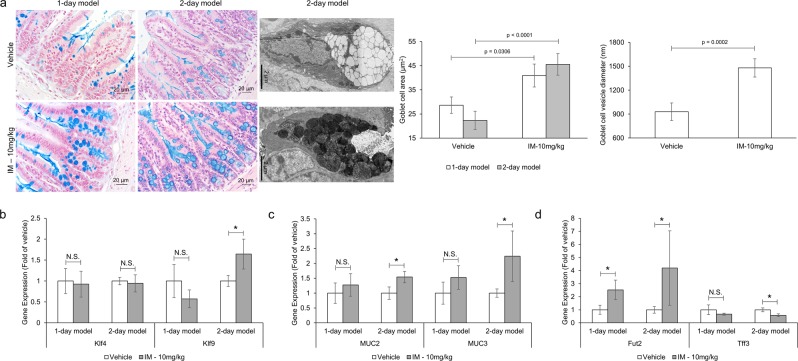
Indomethacin disrupts goblet cell function. Mice (n = 5–12/group) were administered indomethacin (10 mg/kg) or vehicle control every 24 h for 1 or 2 days. Twenty-four hours post last treatment, small intestinal sections were evaluated for secretory vesicles and cell size. Gene expression of goblet cell markers were evaluated from mucosal scraping via qPCR. (**a**) Representative images (left panel) and quantification (right panel) of goblet cell and vesicle size via alcian blue staining and electron microscopy, respectively. Relative mRNA expression of goblet cell maturation markers (**b**), mucin production (**c**) and mucin composition (**d**) from small intestine mucosal scrapings of indomethacin- and control-treated mice. Values and error bars represent the average and 95% confidence intervals, respectively. IM: indomethacin, *p < 0.05; N.S. = no statistical significance.

Autophagy is indispensable for intestinal homeostasis. Its inhibition has been shown to compromise the integrity of the mucosal barrier promoting the invasion and dissemination of opportunistic pathogens leading to an uncontrolled inflammatory response^28,29^. The impact of NSAID-induced autophagy inhibition would be most prominent in secretory IECs where autophagic-dependent functions essential for intestinal homeostasis have been well established. Specifically, in Paneth and goblet cells, autophagy has been shown to regulate the excretion of antimicrobial proteins/peptides that form the mucus layer restricting the direct contact of IECs with luminal material^28,29^. Without autophagy, these cells cannot properly secrete mucin and antimicrobial compounds, causing structural abnormalities and cell enlargement, respectively, and a defective mucosal barrier^28,30^. To examine the effects of NSAIDs on goblet cells, small intestinal sections from control or indomethacin-treated animals were stained with Alcian blue for highly glycosylated proteins, such as mucin, or analyzed via electron microscopy for vesicle architecture and size^31^. In addition, the effect of NSAIDs on goblet cell maturation, mucin production and mucus composition were assessed through gene expression. Briefly, mice were administered a daily dose of indomethacin or vehicle control for 1 or 2 days. Twenty-four hours post last treatment, mice were euthanized and samples were harvested. Mice treated with two doses of indomethacin (2-day model) displayed a stronger inflammatory response and thus a more pronounced phenotype (Fig. S1). Consequently, the 2-day model was used to allow for greater discrimination between the control and indomethacin treated groups at the intracellular level. Indomethacin-treated animals displayed increased goblet cell and vesicle size as determined by Alcian blue staining and electron microscopy, respectively (Fig. 6a). Significant differences in the goblet cell maturation marker Klf9 were observed but there were no significant differences in Klf4 (Fig. 6b). The expression of membrane-bound (i.e. MUC3) and secreted (i.e. MUC2) mucins were significantly upregulated in the ileal mucosa following a two-day indomethacin treatment, which may further contribute to the observed goblet cell enlargement (Fig. 6c). Genes known to alter the composition of the mucus layer were differentially expressed between the control and indomethacin-treated animals suggesting that mucus layer properties are altered in the presence of indomethacin (Fig. 6d). Specifically, the expression of Fut2, an enzyme responsible for addition of fucose residues to carbohydrate moieties, was upregulated in IM-treated animals. In line with this observation, small intestinal sections from indomethacin-treated mice displayed increase UEA-1 staining, implying an increase in fucose residues (Fig. S2). Tff3, a small peptide expressed by goblet cells and known to facilitate intestinal restitution and mucosal protection, was downregulated by indomethacin. Taken together, these data suggest that indomethacin alters the secretion and properties of the ileal mucus layer of the ileum.

**Figure 7:**
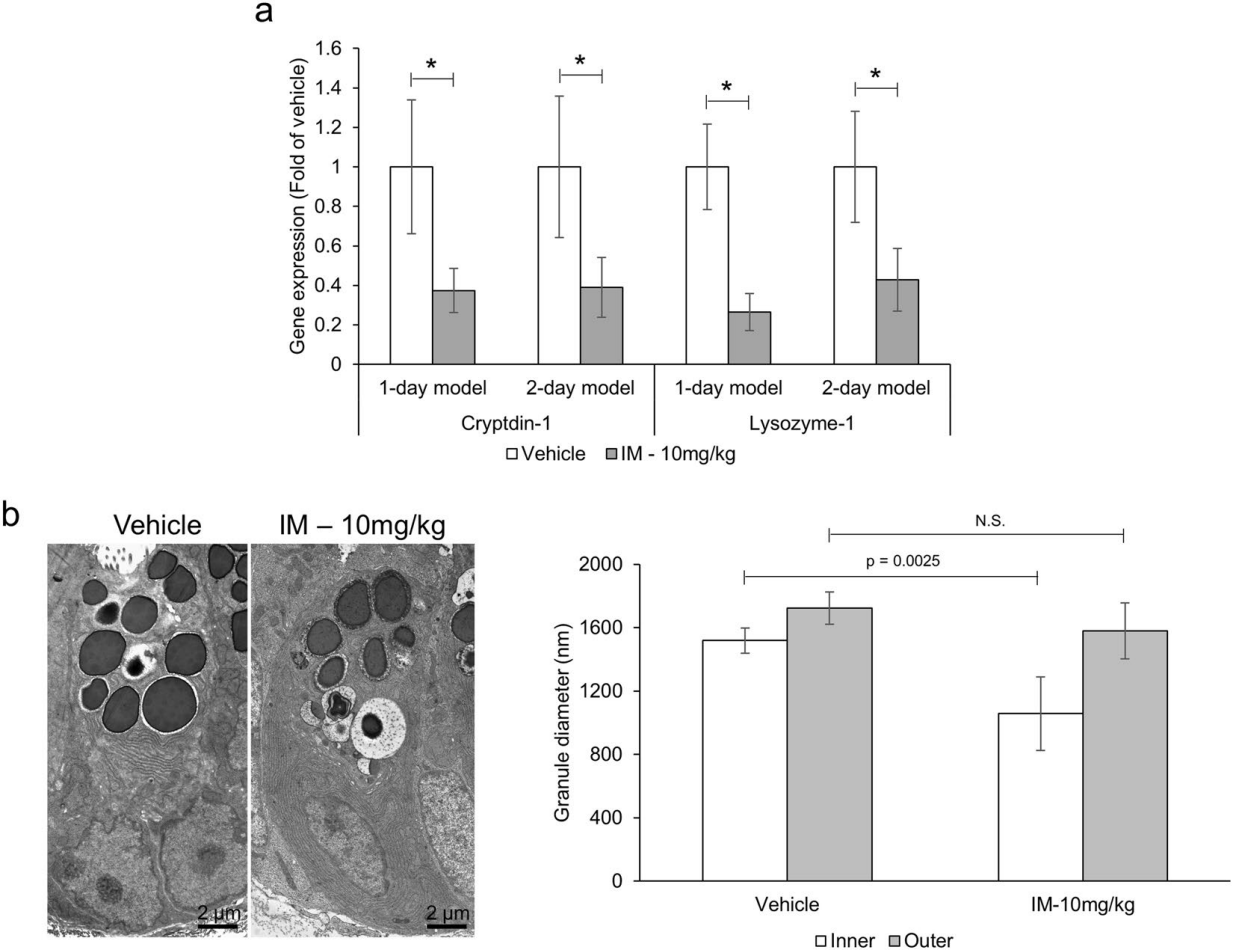
Indomethacin renders Paneth cells dysfunctional. Mice (n = 5–12/group) were administered indomethacin (10 mg/kg) or vehicle control every 24 h for 1 or 2 days. Twenty-four hours post last treatment, small intestinal sections and mucosal scrapings were evaluated for Paneth cell granule size and endoplasmic reticulum thickness and antimicrobial peptide expression, respectively. (**a**) Relative mRNA expression of antimicrobial peptides from small intestinal tissue of indomethacin- and control-treated mice (n = 5–12/group). (**b**) Representative images and quantification of Paneth cell granule size from intestinal sections of indomethacin- and control-treated mice (note, images shown are representative of Paneth cell granules but not of ER appearance, as the magnification and direction of slice are not appropriate to evaluate ER layer thickness). Values and error bars represent the average and 95% confidence intervals, respectively. IM: indomethacin, *p < 0.05; N.S. = no statistical significance.

To assess the effects of indomethacin on Paneth cells, the expression of the antimicrobial peptides, lysozyme and cryptdin-1, collected from mucosal scrapings were measured via qRT-PCR. Concomitantly, the structure of secretory granules and thickness of the endoplasmic reticulum from Paneth cells of indomethacin- and control-treated mice was evaluated through electron microscopy. Both antimicrobial peptides examined were significantly reduced in indomethacin-treated mice compared to vehicle control (Fig. 7a). This was further supported by the abnormal morphology of secretory granules and enlarged endoplasmic reticulum in Paneth cells of indomethacin-treated animals compared to control (Figs 7b and S3).

**Figure 8:**
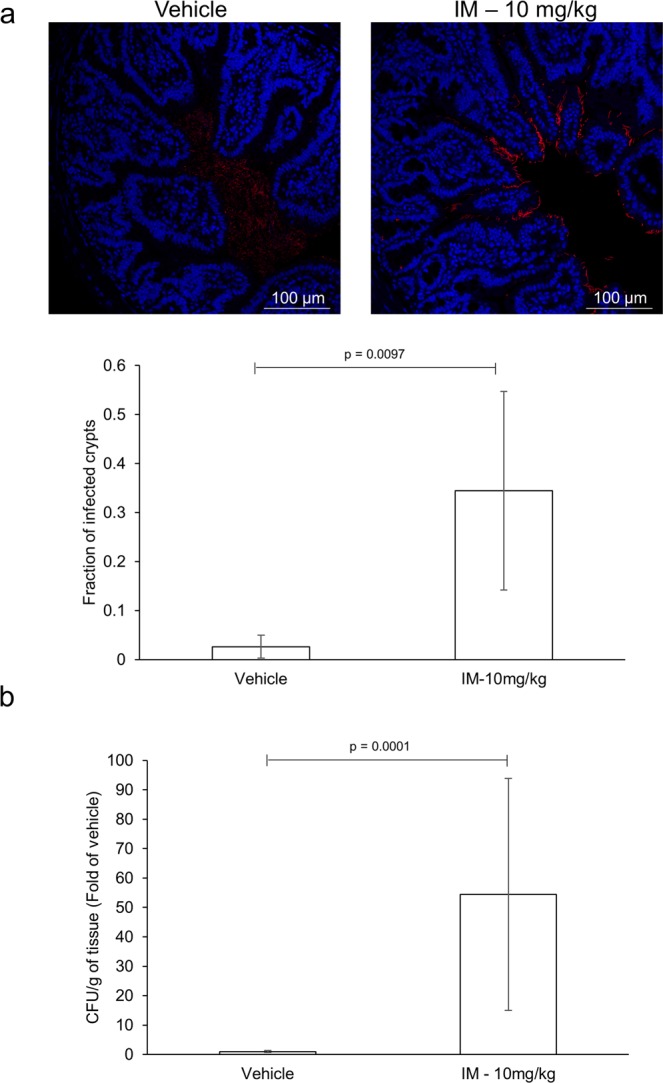
Indomethacin promotes invasion of luminal material. Mice (n = 6–12/group) were administered indomethacin (10 mg/kg) or vehicle control every 24 h for 2 days. Twenty-four hours post last treatment, small intestinal sections were stained for bacteria using universal probe (EUB338). Dissemination of bacteria was determined by quantitative culture of liver samples from the two-day model. (**a**) Representative images and fraction of infected crypts from small intestinal sections of indomethacin- and control-treated mice. (**b**) Quantitative culture of liver samples. IM: indomethacin.

The mucus layer functions to limit bacterial contact with the epithelium and its systemic translocation. Depletion and/or compositional changes to the mucus layer can allow direct contact, increase pathogenicity/adhesion and invasion of luminal material that can disseminate systemically. To evaluate the effects of indomethacin on barrier function, penetration of bacteria into the intestinal mucosa was examined via fluorescent *in situ* hybridization (FISH). Indomethacin-treated animals displayed increase penetration of luminal contents into the villus gap, with some bacteria residing in the crypts of the small intestine (Fig. 8a). To examine bacterial translocation, liver samples were cultured. An increase bacterial load was cultured from the livers of indomethacin-treated animals compared to control (Fig. 8b). Collectively, these results suggest that indomethacin treatment compromises the integrity of the mucus layer.

**Figure 9:**
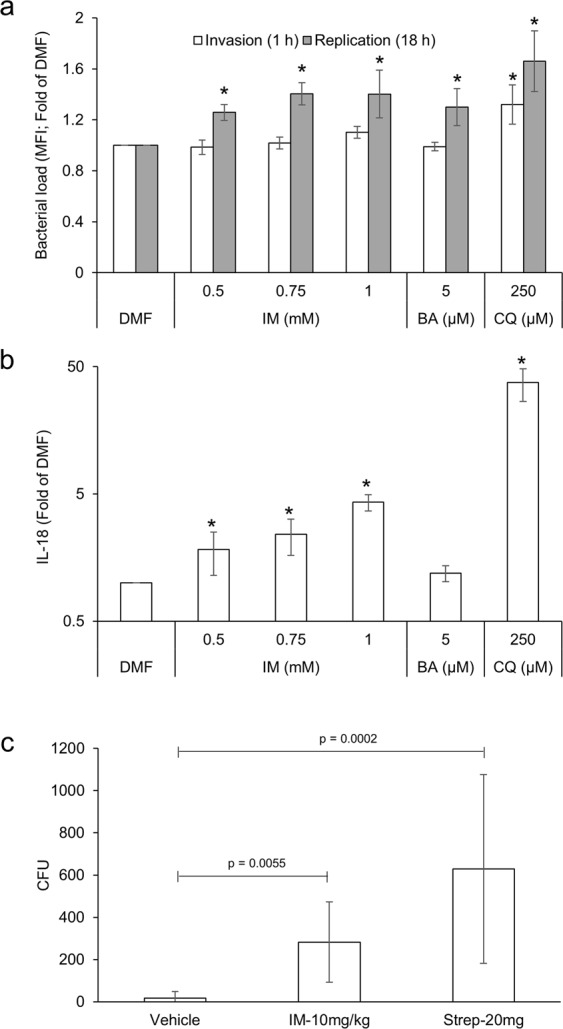
Indomethacin prevents clearance of invading microbes leading to a heightened inflammatory response. (**a**) YAMC cells were infected with *Salmonella* (MOI 100) for 30 minutes, followed by addition of the appropriate compound. At the indicated time point, bacterial load was determined via flow cytometry. (**b**) YAMC cells were infected with *Salmonella* (MOI 100) for 30 minutes, followed by addition of the appropriate compound. Eighteen hours post infection, the concentration of IL-18 in the supernatant was measured and normalized to the number of cells per well. (**c**) Mice (n = 6/group) were administered indomethacin (10 mg/kg), streptomycin or vehicle control. The next day, mice were inoculated with 1 × 10^8^ CFU of *Salmonella* Typhymurium. Twenty-four hours post infection, quantitative culture of viable bacteria in the Peyer’s patch was performed. Values and error bars represent the average and 95% confidence intervals, respectively. IM: indomethacin; Strep: streptomycin, *p < 0.05.

In the event of microbial invasion, IECs exploit autophagy as a means of bacterial clearance^32,33^. Cells deficient in autophagy have been shown to accumulate higher levels of intracellular pathogens, including *Salmonella*, leading to increased secretion of pro-inflammatory cytokines and cell death^34^. To evaluate the effect of NSAIDs on *Salmonella* clearance and subsequent inflammatory response, a gentamicin protection assay was performed. Since indomethacin displayed the strongest autophagic flux inhibition, it was selected as representative of all NSAIDs. Briefly, YAMC cells were infected with *Salmonella* for 30 minutes, followed by exposure to increasing concentrations of indomethacin, positive controls bafilomycin and chloroquine, or DMF vehicle. After 1 or 18 h, the intracellular bacterial load was measured. Concomitantly, the concentration of secreted IL-18 in the supernatant was quantified via ELISA. Cells treated with indomethacin displayed a dose-dependent increase in intracellular bacterial load and enhanced secretion of IL-18 compared to vehicle control, similar to positive controls, bafilomycin and chloroquine (Fig. 9a,b). To confirm our *in vitro* observations, the ability of NSAID-treated mice to clear *Salmonella* after NSAID administration was examined. Briefly, mice (n = 6/group) were administered a single dose of indomethacin (10 mg/kg) 24 h prior to *Salmonella* inoculation. As positive and negative controls, 20 mg of streptomycin and 0.5% CMC/5% DMF, respectively, were used. Forty-eight hours after drug administration, mice were euthanized and samples harvested. Indomethacin- and streptomycin-treated mice displayed higher *Salmonella* loads in all locations examined including Peyer’s patch, cecal contents, and feces compared to vehicle control (Figs 9c and S4), underscoring the importance of autophagy on bacterial clearance. Collectively, these results suggest that NSAID-induced autophagy inhibition disrupts IEC processes essential for host-microbial homeostasis.

## Discussion

The present study demonstrates that multiple classes of NSAIDs inhibit autophagic flux *in vitro* by reducing the area and/or distribution of functional lysosomes in IECs. These findings were recapitulated *in vivo*, with the NSAID indomethacin promoting the accumulation of autophagy substrates in conjunction with reduced staining of lysosomal marker LAMP-1 in murine small intestinal tissue. Indomethacin also disrupted well-characterized IEC processes dependent on autophagy and essential for the symbiotic host-microbiota relationship. Thus, understanding how NSAIDs modulate autophagy is critically important to determine its impact on NSAID enteropathy. In the absence of this mechanistic information, the contribution of autophagy in NSAID-induced intestinal injury remains elusive.

We demonstrate that treatment of IECs with multiple classes of NSAIDs resulted in an increase in LC3-II/I ratio and p62, indicative of autophagic flux inhibition. The accumulation of both autophagy substrates implies that NSAIDs inhibited a later step, likely after autophagosome formation and prior to cargo degradation. Indeed, mechanistic studies revealed that NSAID-treated IECs exhibit reduced the area of LAMP-1 and LysoTracker® positive vesicles that clustered near the perinuclear region. Lysosomes are required in the final step of autophagy, where fusion of autophagosomes and lysosomes leads to encapsulated cargo degradation by lysosomal enzymes^35^. Thus, the concomitant accumulation of autophagosome substrates and reduced lysosomal staining, may be a potential mechanism underlying NSAID-induced autophagy inhibition. The exact mechanism by which NSAIDs reduced lysosome area is unclear. Lysosomal vesicles are derived from the maturation of endosomes, and thus, it may be possible that NSAIDs hindered endosomal trafficking and lysosomal biogenesis^36^. Alternatively, NSAIDs have been proposed to act as cationic amphiphilic drugs, that can interact with vesicles and change their physico-chemical properties^37^. The similar pattern observed with both the membrane-associated LAMP-1 and the pH-sensitive probe LysoTracker® suggests that NSAIDs reduce the area of lysosomes and not their acidification. This is further supported by differences in lysosome distribution between NSAID-and CQ-treated cells, where lysosomal pH is altered. Moreover, induction of autophagy by nutrient starvation promotes accumulation of lysosomes near or at the perinuclear region, where proper acidification and enhanced substrate degradation is observed, suggesting that NSAID-induced altered lysosome distribution should not affect their buffering capacity^38^. These observations are in agreement with Vallecillo-Hernández *et al*., where treatment of gastric cancer cells (AGS cells) with indomethacin disrupted autophagic flux by inducing lysosomal dysfunction^15^. Contrary to our findings, inhibition of autophagy was attributed to a reduction in Lysotracker® probe intake accompanied by centrifugal movement of lysosomes to the cell periphery, suggestive of lysosome basification and not a reduction in the area of lysosomal vesicles. Unlike cancer-derived cell lines (*e*.*g*., AGS) that commonly display altered autophagy machinery for the benefit of the cell survival, YAMCs might provide a more accurate representation of how NSAIDs modulate autophagy in “naïve” cells^16^. A similar response was documented *in vivo*, where mice administered a single dose of indomethacin displayed accumulation of autophagy substrate p62 and an increase ratio of LC3-II/I accompanied by a reduction in LAMP-1 staining compared to vehicle control. Collectively, these results suggest that NSAIDs disrupt autophagic flux, likely, by reducing the area and/or distribution of functional lysosomes, preventing the final step(s) of autophagy.

Intestinal homeostasis is maintained by the coexistence of commensal microorganisms with host cells through a physicochemical barrier, namely the mucus layer. Although the commensal microbiota is considered harmless, upon mucosal damage these cells can act as opportunistic pathogens and contribute to the pathology of GI disorders^39^. Autophagy and microbial presence (sensing) have been shown to stimulate the secretion of mucin and antimicrobial peptides (AMPs), the main building blocks of the mucus layer^28,40,41^. In the absence of autophagy, AMPs and mucin are trapped inside Paneth and goblet cells, respectively, compromising the integrity of the mucosal barrier^28,30^. Hence, mucin-deficient mice develop spontaneous inflammation and display higher susceptibility to pathogens, including *Salmonella*^42,43^. We observed a significant enlargement in the size of goblet cells and secretory vesicles in mice treated with indomethacin, as gauged by the accumulation of Alcian blue- positive staining (*i*.*e*., mucin) and electron microscopy, respectively. Similarly, the secretory vesicles and granules of goblet and Paneth cells were significantly enlarged and disfigured, respectively upon indomethacin treatment. These findings suggest entrapment and accumulation of mucus building blocks in these cells. Enzymes/peptides know to alter the composition of the mucus layer were also found to be differentially expressed in indomethacin treated animals implying that both the thickness and properties of the mucus layer may be affected in the presence of NSAIDs. Indomethacin treated mice displayed enhanced penetration and invasion of luminal material into the crypts as well as higher bacterial counts in the liver, suggesting that modulation of the mucus layer by indomethacin promotes direct contact and invasion of microbial cells, ultimately reaching nearby organs. This might also explain the microbial dysbiosis associated with NSAID enteropathy, whereby changes in mucus composition/thickness may favor the outgrowth of mucin degrading and tethering microbes and pathogens^44–46^. IECs and mice exposed to indomethacin also presented higher loads of intracellular *Salmonella* compared to their relative controls. Because clearance of intracellular pathogens is also controlled by autophagy, once invaded, IECs can no longer clear these opportunistic pathogens inciting a heightened inflammatory response. This might partially explain why germ-free or antibiotic-treated rodents are resistant to NSAID-induced small bowel injury^35–38^. Since *Salmonella* is known to exploit inflammation and microbial dysbiosis to promote its own colonization and outcompete resident microflora^47,48^, the dysbiotic state and inflammatory response associated with NSAID administration might also contribute to the increased *Salmonella* burden^49,50^. However, the similar phenotype observed *in vitro*, in the absence of a microbiota component, confirms that NSAID-induced autophagy inhibition is likely to play a role. Collectively these results suggest that NSAID-induced autophagy inhibition compromises the integrity of the mucus layer. This combined with the inability of IECs to clear invading pathogens and coordinate the responses of sub-epithelial cells, will lead to an uncontrolled inflammatory response limiting the repair of the mucosa thereby disrupting intestinal homeostasis.

Understanding how NSAIDs modulate autophagy will help to elucidate its role on intestinal homeostasis and NSAID enteropathy, a disease afflicting both humans and animals. Unlike the artificial induction of inflammation/injury by chemicals (*e*.*g*., DSS colitis), models of NSAID enteropathy represent a natural and relevant alternative to study GI-associated diseases with similar clinical features (*e*.*g*., autophagy deficiency, microbial dysbiosis, uncontrolled inflammatory response). Specifically, inflammation and defects in autophagy are hallmarks of other intestinal diseases, including IBD, allowing for examination of pathways common to numerous GI diseases and disorders. The current study strengthens evidence of the important role of IEC autophagy in NSAID enteropathy. It also raises more questions including: (1) mechanism by which NSAIDs decrease the area of lysosome population in IECs; (2) the effects of NSAID-induced inhibition of autophagy on the different IEC types found in the intestinal mucosa; and, (3) correlation of NSAID-induced inhibition of autophagy with other known contributors to the pathophysiology of NSAID enteropathy (*e*.*g*., microbial dysbiosis). On the basis of our results, we aim to investigate the mechanism by which NSAID treatment of IECs reduces the area and distribution of lysosomal vesicles available for cargo degradation and the extent of autophagy inhibition by NSAIDs on immune cells. The outcomes of these studies should provide a more comprehensive understanding on the impact of autophagy on the pathogenesis of NSAID enteropathy and other inflammatory intestinal conditions.

## Materials and Methods

### Cell culture

YAMC cells were kindly provided by Dr. Robert Whitehead^16^. Unless otherwise stated, YAMC cells were cultured in RPMI 1640 media containing GlutaMAX, Hepes (Thermo Fisher Scientific, Waltham, MA) and supplemented with 5% fetal bovine serum (Thermo Fisher Scientific), ITS (Corning, Tewksbury, MA) and mouse interferon gamma (Sigma-Aldrich, St.Louis, MO).

### Autophagic flux

To determine the effect of NSAIDs on autophagic flux, YAMC cells were seeded in 6-well plates (8.5 × 10^5^ cells/well) and incubated under non-permissive conditions at 37 °C/5% CO_2_. The next day, media was replaced with the appropriate drug and returned to the incubator. Twenty-four hours post drug addition, cells were washed once with DPBS. For flow cytometry, cells were detached and transferred to a centrifuge tube for staining. For western blot, 250 μL of radioimmunoprecipitation assay buffer (RIPA; 150 mM sodium chloride, 1.0% NP-40, 0.5% sodium deoxycholate, 0.1% sodium dodecyl sulfate (SDS), 50 mM Tris, pH 8.0) supplemented with protease inhibitor cocktail (Sigma Aldrich) were directly added to the well. Plates were incubated at 4 °C for 10–20 minutes. Lysates were transferred to a tube and centrifuged at 14,000 × g for 15 min. Protein concentration was measured from the supernatant using the bicinchoninic acid assay (Thermo Fisher Scientific).

### *Salmonella* infection assays

*Salmonella* infection assays were carried out as previously described^51,52^. Briefly, a fresh colony of *Salmonella enterica* serovar Typhimurium (ATCC 14028) was used to inoculate 2 mL of Luria broth (LB; BD Biosciences, San Jose, CA) and grown for 16–18 h at 37 °C and 220 rpm. The next day, the overnight culture was diluted 40-fold in 10 mL of fresh LB and grown for 3.5 h at 37 °C and 220 rpm. Cultured bacteria were washed once with DPBS, resuspended to the desired optical density and used to infect previously seeded YAMC cells (8.5 × 10^5^ cells/well in 6-well plates) at a multiplicity of infection (MOI) of 100 under non-permissive conditions. Thirty minutes post infection, cells were washed and replaced with gentamicin (100 μg/mL) containing media and the appropriate drug. One hour later, media was replaced with a lower gentamicin concentration (10 μg/mL) and the appropriate treatment. At the indicated time points, cells were washed once with DPBS, detached and transferred to a centrifuge tube for staining.

### IL-18 secretion

YAMC cells seeded in 96-well plates at 4 × 10^4^ cells/well, were infected with *Salmonella* at an MOI of 100 and treated with the appropriate compound as described above (*Salmonella* infection assays). Eighteen hours post infection, the concentration of secreted IL-18 in the supernatant was measured via ELISA (ab218808, Abcam Cambridge, MA) as per manufacturer’s protocol. IL-18 concentrations were normalized to cell number as assessed by the Janus green assay^53^.

### Western Blot analysis

Protein lysates (6.5–9 μg for cells and 25 μg for small intestine mucosal scrapings) in 1X SDS/β-mercapthoethanol buffer were resolved on a 4–20% TGS stain free gel (BioRad, Hercules, CA) and electrotransferred onto a polyvinylidene difluoride transfer membrane (Thermo Fisher Scientific). Western blot analysis was performed using rabbit anti-LC3A/B (1:1000; Cell Signaling Technology #4108, Danvers, MA), rabbit anti-SQSTM1/p62 (1:1000; Cell Signaling Technology #5114) or rabbit anti-GAPDH (1:1000; Cell Signaling Technology #5174) and horseradish peroxidase-conjugated goat anti-rabbit (1:2000; Cell Signaling Technology #7074) antibodies. Protein bands were visualized by chemiluminescence using a ChemiDocTouch Imaging System (BioRad). Bands were quantified using the ImageLab software Version 5.2.1.

### Flow cytometry

Collected cells were fixed and permeabilized with CytoFix/CytoPerm solution (BD Biosciences) for 1 h at 4 °C. Cells were washed once with Perm/Wash buffer (BD Biosciences) and incubated in the same buffer for 30 minutes at 4 °C. After incubation, cells were centrifuged, stained with rabbit anti-SQSTM1/p62 (1:100; Cell Signaling Technology #5114) or *Salmonella* O antiserum group B (1:250; BD Biosciences #229481) overnight at 4 °C followed by Alexa Fluor 488-conjugated anti-rabbit (1:500; Cell Signaling Technology #4412) antibodies for 1 h at room temperature. Samples were analyzed using a BD FACScan flow cytometer (BD Biosciences).

For LysoTracker® experiments, YAMC cells were seeded in 24-well plates (1.9 × 10^5^ cells/well) and incubated under non-permissive conditions (37 °C, 5% CO_2_). The next day, media was replaced with the appropriate drug treatment and cells were returned to the incubator (37 °C, 5% CO_2_). Twenty-four hours later, cells were stained with 50 nM LysoTracker® Red DND-99 (Invitrogen, Carlsbad, CA) for 30 minutes at 37 °C. Cells were detached from the plates, centrifuged and resuspended in PBS. Samples were analyzed using a BD FACScan flow cytometer (BD Biosciences).

### Microscopy

For LysoTracker® experiments, YAMC cells were seeded in 2-well chamber coverglass (2.1–5.1 × 10^5^ cells/well) and incubated under non-permissive conditions (37 °C, 5% CO_2_). Treatments were added 24 h prior to image acquisition. Lysosomal vesicles and DNA were stained with 50 nM LysoTracker® Red DND-99 (Invitrogen) and Hoechst 33342 (Invitrogen) for 30 and 15 minutes, respectively, at 37 °C. Cells were imaged using a Zeiss LSM 780 confocal microscope. To quantify the effect on lysosomal vesicles, the area of Lysotracker® positive vesicles per field was measured and normalized to that of DMF control.

For LAMP-1 staining in cells, YAMC cells were seeded in 8-well chamber coverglass (3.2–7.7 × 10^4^ cells/well) and incubated under non-permissive conditions (37 °C, 5% CO_2_). The next day, media was replaced with the appropriate treatment. Twenty-four hours post drug addition, cells were washed, fixed with 4% paraformaldehyde, blocked with 5% normal goat serum (NGS) and stained with mouse anti-LAMP1/CD107a (1:400; Thermo Fisher #MA1-25620) at 4 °C overnight followed by Alexa Fluor 488-conjugated anti-mouse (1:600; Cell Signaling Technologies #4408) for 1 h at room temperature. Cells were imaged using a Zeiss LSM 780 confocal microscope. To quantify the effect on lysosomal vesicles, the area of LAMP-1 positive vesicles per field was measured and normalized to that of DMF control.

For LAMP-1 staining in formalin-fixed paraffin embedded (FFPE) tissue, sections (5 µm thickness) were incubated at 55 °C for 15 min, deparaffinized (D-limonene) and subjected to antigen-retrieval (Diva Decloaker, 100 °C for 10 min). Sections were permeabilized with DPBS/1% NGS/0.4% Triton, blocked with DPBS/5% NGS/0.4% Triton and stained with rat anti-LAMP-1 (3.5 μg/mL; DSHB 1D4B) and rabbit anti-p62 (1:125; Novus Biologicals #NBP1-49956, Littleton, CO) at 4 °C overnight followed by Alexa Fluor 488-conjugated anti-rat (1:125, Invitrogen #A11006) and Alexa Fluor 594-conjugated anti-rabbit (1:500; Cell Signaling Technologies #8889) in DPBS/1% NGS/0.4% Triton for 1–2 h at room temperature. Tissues were imaged using a Zeiss LSM 780 confocal microscope.

For quantification of goblet cell area, FFPE or methacarn fixed tissue sections (5 µm thickness) were stained with Alcian blue. Ten representative images (400x) per tissue were collected using an Olympus BX40 bright field microscope with an 18MP microscope camera (AmScope) and ToupView software v3.7.7934 (ToupTek, Zhejiang, China. Using the TopView software, boundaries were drawn around each cell and used to calculate cell area (4912 pixels = 230 μm using a 1 mm microscope stage calibration slide). Goblet cells without defined borders or in contact with field margins were not analyzed.

For bacteria visualization, methacarn fixed tissue sections (5 µm thickness) were stained with EUB-338 probe conjugated to AlexaFluor 594 as previously described^54,55^. Tissues were imaged using a Zeiss LSM 780 confocal microscope. To quantify the fraction of infected crypts, eight representative images per tissue were examined for bacteria positive per total number of crypts in the field.

For electron microscopy analysis, ileal sections were prepared similarly to those previously described^56^. Approximately 1 cm segments of fresh mucosal tissues were split longitudinally, pinned flat in sylgard-coated petri dishes with the mucosal side up, and immersed in an aldehyde fixative solution (1.25% glutaraldehyde and 4% paraformaldehyde in 0.1 M cacodylate buffer, pH 7.4) for 1.5 h on a shaker at room temperature. The tissues were then post-fixed in 1% osmium tetroxide solution in 0.1 M cacodylate buffer for 1 h on a shaker at room temperature, and stained with saturated uranyl acetate for 1 hr on a shaker at room temperature. After dehydration in increasing concentrations of ethyl alcohol (30%, 50%, 80%, 95%, and 100%), the tissues were infiltrated with propylene oxide and embedded in Eponate 12. The tissues were sectioned (~100 nm thick) using a Leica EM UC6 ultramicrotome and Diatome diamond knife, and the sections were stained with uranyl acetate and Reynold’s lead citrate. Paneth cells and goblet cells were imaged using an FEI Morgagni 268 transmission electron microscope equipped with an Imageview III CCD camera. Photoshop software package was used to adjust the brightness and contrast of images and arrange the figure panels.

Paneth cell granule diameters, Goblet cell vesicle diameters and endoplasmic reticulum thicknesses were measured using the ‘Analysis-Measure’ function in the Image J software package. To account for irregularities in shape, the diameters of Paneth cell granules (inner and outer diameters) and Goblet cell vesicles were each expressed as the average of diameter measurements along 4 axes (each axis was rotated by 45-degree increments). Further, the thickness of an endoplasmic reticulum layer was expressed as the average of 5 different measurements taken at random positions along its length.

### *In vivo* studies

Animal protocols were approved by the Texas A&M University’s Institutional Animal Care and Use Committee in accordance with appropriate institutional and regulatory bodies’ guidelines (AUPs 2017-0140, 2017-0142, 2017-0290). Animals were handled and treated as previously described^57^. Briefly, 8- to 10-week-old, specific-pathogen-free C57BL/6J mice were purchased from Envigo and allowed to acclimate for 1–2 weeks. Mice were fed standardized laboratory rodent diet (Teklad rodent diet, Envigo product number 8604) and sterile water ad libitum. Mice were randomly divided into 2 groups (n = 3–6 mice/group/cage): indomethacin 10 mg/kg or vehicle control and rehoused according to group assignment. To prepare treatments, a stock solution of indomethacin in DMF or NMP was diluted in DPBS to the desired final concentration. Indomethacin and vehicle control (200 µL of 2.4% DMF or 0.5% NMP) were administered via oral gavage. Feces were collected before, 24 and 48 h after gavaging by placing individual animals in ventilated specimen cups until they passed feces and immediately flash frozen at −80 °C. Twenty-four hours post last gavage, mice were euthanized via CO_2_ asphyxiation. The last third of the small intestine was collected, opened longitudinally, washed with cold PBS supplemented with protease and phosphatase inhibitors and cut in half. The distal half was fixed i) in methacarn or ii) 10% formalin and Swiss-rolled, paraffin-embedded, and stained with hematoxylin and eosin (H&E), Alcian blue or used for immunofluorescence studies. H&E stained sections were scored for intestinal inflammation by a blinded board-certified anatomic veterinary pathologist as previously described^23^. For the proximal half, mucosal scrapings were collected and frozen at −80 °C for subsequent gene expression analysis and protein quantification.

For *in vivo Salmonella* infection assays, mice (12- to 15-week-old, n = 6/group) were oral gavaged with 200 μL of NSAID indomethacin (10 mg/kg in vehicle), streptomycin (20 mg in vehicle) or vehicle control (DPBS/5% DMF/0.5% carboxymethylcellulose). Twenty-four hours later, mice were inoculated with 200 μL of 5 × 10^8^ CFU/mL *Salmonella enterica* serovar Typhimurium (ATCC 14028) in DPBS. Twenty-four hours post inoculation, feces were collected as described above and transferred into a clean tube filled with PBS. Mice were euthanized via CO_2_ asphyxiation. A single Peyer’s patch (PP) closest to the cecum, and cecal contents were collected and transferred into a clean tube containing PBS. Fecal and cecal samples were homogenized by vortexing, diluted as necessary and plated in LB-Agar supplemented with 25 μg/mL of nalidixic acid. PP was ground, diluted as necessary and plated in LB-Agar supplemented with 25 μg/mL of nalidixic acid. Plates were incubated overnight at 37 °C. Colonies were counted the next day and normalized to sample weight.

### Calprotectin ELISA

Fecal calprotectin levels were measured via ELISA as per manufacturer’s protocol with minor modifications. Briefly, ~100 mg of feces was added to 4.9 mL of extraction buffer (0.1 M Tris, 15 mM NaCl, 1 M urea, 1 mM CaCl_2_, 0.1 M citric acid, 0.5% BSA, 0.25% gentamycin, pH 8), vortexed and centrifuged at 3000 × g for 5 minutes. Supernatant was collected, diluted (250–25,000-fold) in 1% BSA/PBS and used to measure calprotectin concentrations. Values are expressed as amount of calprotectin per gram of feces.

### qRT-PCR

RNA from intestinal scrapings was extracted using the EZNA Total RNA kit (Omega Bio-Tek) as per manufacturer’s protocol. Gene expression levels were measured via Real-Time Quantitative Reverse Transcription PCR (qRT-PCR) (qScript One-Step Kit, Quanta Biosciences, Gaithersburg, MD) using the primers shown in Table 1.

**Tablee 1:** Gene name and primers used in this manuscript.

Gene	Primer	Sequence (5′→3′)
mIL-1β	Sense	AAA CCT TTG ACC TGG GCT GTC
	Antisense	TCA CAG AGG ATG GGC TCT TCT
mTNFα	Sense	CCA TGA GCA CAG AAA GCA TGA TC
	Antisense	GCC ATT TGG GAA CTT CTC ATC C
mKlf4	Sense	GCG AAC TCA CAC AGG CGA
	Antisense	CAT GTG TAA GGC AAG GTG GTC C
mKlf9	Sense	CCA TTA CAG AGT GCA TAC AGG TGA AC
	Antisense	GGAACTCGGTGTGACGC
mMUC2	Sense	CTCTGTGCCAAGGAAGGTGTCT
	Antisense	CAG GTC CCA CAC ATC CAC A
mMUC3	Sense	CGTAGAGATAGAGCCGACAGTC
	Antisense	CAC GAT ACT GCC TTT GCT CAG A
mTff3	Sense	GCC TGT CTC CAA GCC AAT G
	Antisense	TCA AAA TGT GCA TTC TGT CTC CTG
mFut2	Sense	ACCACAGCCAGAAGAGCC
	Antisense	GAA TTG ATC GTG AAT ATG CCC TGC
mGAPDH	Sense	TGT CAA GCT CAT TTC CTG GTA TGA CA
	Antisense	GAGTTGGGATAGGGCCTCTCTT

### Bacterial dissemination

To measure bacterial dissemination, the right lateral or medial liver lobe was harvested, dipped in ethanol followed by PBS, ground in fresh PBS and plated in LB-Agar under aerobic conditions for 24 to 48 h. Counted colonies were normalized to tissue weight. For plates with no visible or too much growth, the number of colonies was set arbitrarily to 1 and 1000, respectively.

### Data analysis

Data were analyzed using S-PLUS statistical software (Version 8.2, TIBCO Inc., Seattle, WA) unless otherwise noted. Outcomes measured at one time-point (*i*.*e*., microscopy, flow cytometry, gene expression, and protein expression) were compared among treatment groups using a generalized linear model with post hoc testing for pairwise differences among groups using the method of Sidak^58^. Data were log_10_- transformed to meet statistical assumptions when needed. For repeated measures (*i*.*e*., fecal calprotectin and *Salmonella* CFU), linear mixed-effects regression was used to account for repeated measures on individual mice (*i*.*e*., mouse modeled as a random effect). When appropriate, data were log_10_-transformed to improve model fit. Post hoc comparisons of pairwise differences among treatments and days were made using the method of Sidak^47^. Microscopic pathology scores were compared with the Wilcoxon rank-sum test. For all analyses, significance was set P < 0.05.

## Supplementary information

Supplementary Information
