# Supplementary material for: NSAIDs disrupt intestinal homeostasis by suppressing macroautophagy in intestinal epithelial cells
Source: Sci Rep. 2019 Oct 10;9:14534. doi: 10.1038/s41598-019-51067-2 (PMC6787209; doi:10.1038/s41598-019-51067-2)
Supplement: Supplementary file 1 — Supplementary Information [file 41598_2019_51067_MOESM1_ESM.pdf]

Title: NSAIDs disrupt intestinal homeostasis by suppressing macroautophagy in intestinal epithelial cells

Ana M. Chamoun-Emanuelli[1], Laura K. Bryan [1], Noah D. Cohen[1], Taylor L. Tetrault[1], Joseph A. Szule [2], Rola Barhoumi [3], Canaan M. Whitfield-Cargile[1*]

[1]Department of Large Animal Clinical Sciences, College of Veterinary Medicine & Biomedical Sciences, Texas A&M University, College Station, Texas, United States of America

[2]Department of Veterinary Pathobiology, College of Veterinary Medicine & Biomedical Sciences, Texas A&M University, College Station, Texas, United States of America

[3]Department of Veterinary Integrative Biosciences, College of Veterinary Medicine & Biomedical Sciences, Texas A&M University, College Station, Texas, United States of America

***Corresponding Author:** Canaan Whitfield-Cargile
Department of Large Animal Clinical Sciences
College of Veterinary Medicine & Biomedical Sciences
Texas A&M University
4475 TAMU
College Station, TX 77843-4475
Cwhitfield@cvm.tamu.edu
Phone: (979) 845-9135; Fax: (979) 847-8863

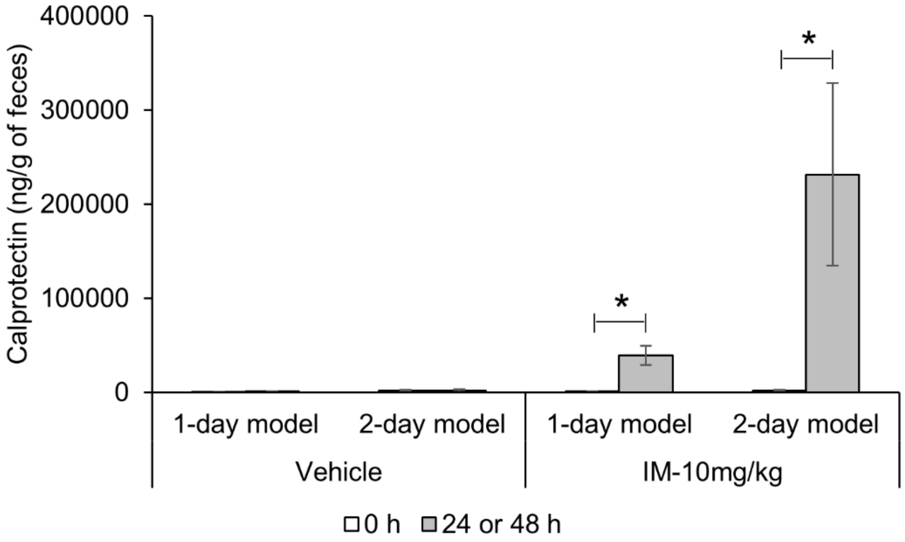

Figure S1. Indomethacin induces small intestinal injury. Mice (n = 6/group) were administered indomethacin (10 mg/kg) or DMF control every 24 h for 1 or 2 days. Twenty-four hours post last treatment, the inflammatory response was evaluated via increase in fecal calprotectin levels. Values and error bars represent the average and 95% confidence intervals, respectively. IM: indomethacin, * = $p < 0.05$

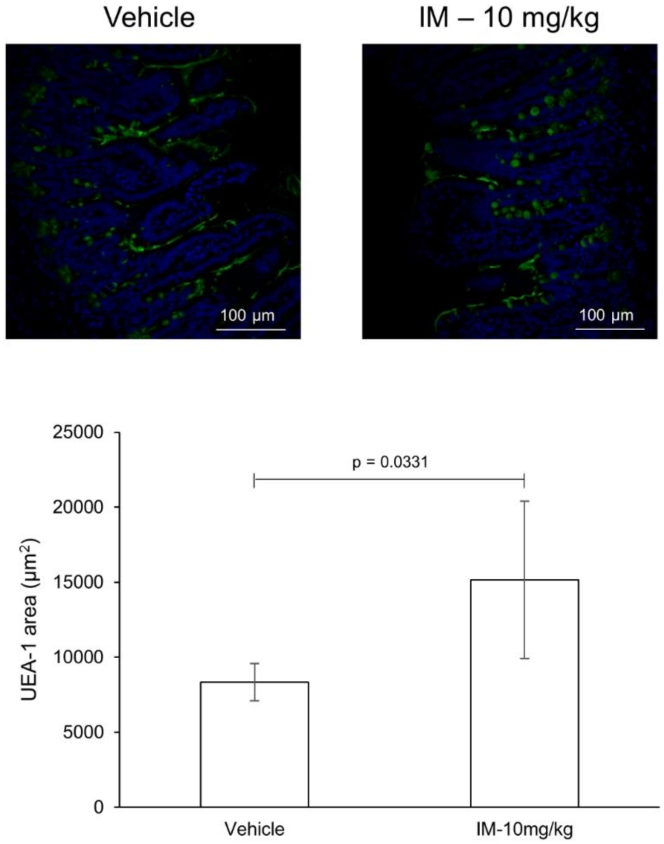

Figure S2. Indomethacin modulates mucin composition. Mice (n = 6/group) were administered indomethacin (10 mg/kg) or DMF control every 24 h for 2 days. Twenty-four hours post last treatment, the presence of fucosylated residues was evaluated via via microscopy. Representative images (top panel) and staining area of UEA-1 (bottom panel) from small intestinal sections of indomethacin- and control-treated mice Values and error bars represent the average and 95% confidence intervals, respectively. IM: indomethacin

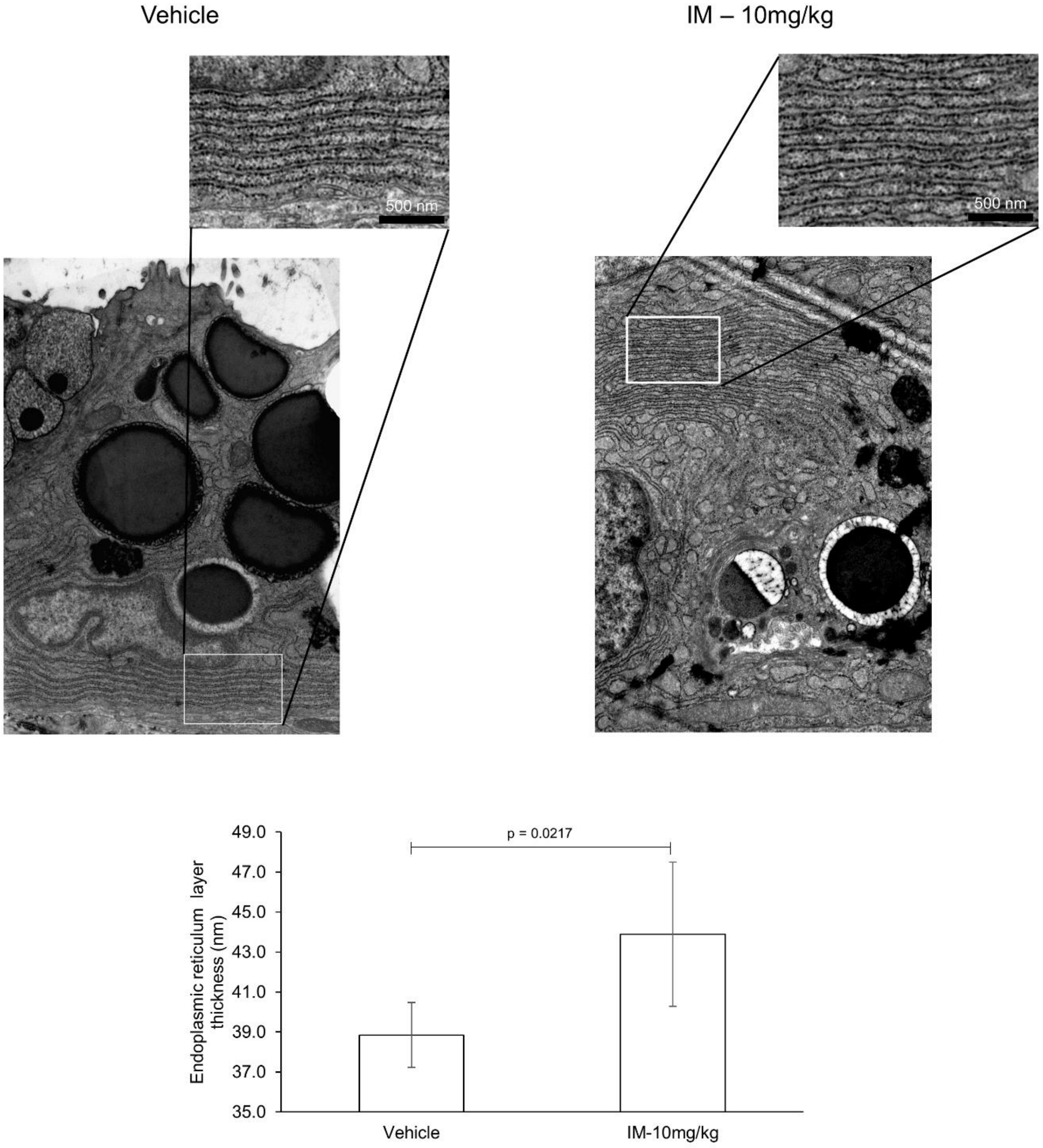

Figure S3. Indomethacin induced endoplasmic reticulum enlargement. Mice (n = 4-6/group) were administered indomethacin (10 mg/kg) or DMF control every 24 h for 2 days. Twenty-four hours post last treatment, the thickness of the endoplasmic reticulum

layers were imaged and analyzed using electron microscopy. Representative images (top panel) and quantification (bottom panel) from small intestinal sections of indomethacin- and control-treated mice. Values and error bars represent the average and 95% confidence intervals, respectively. IM: indomethacin

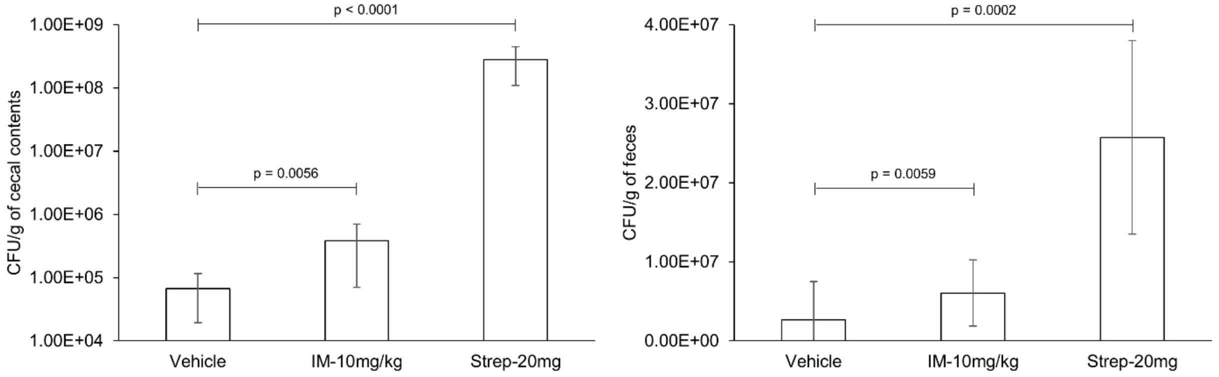

Figure S4. Indomethacin prevents bacterial clearance. Mice (n = 6/group) were administered indomethacin (10 mg/kg), streptomycin or vehicle control. The next day, mice were inoculated with 1 x 10^8 CFU of *Salmonella* Typhymurium. Twenty-four hours post infection, quantitative culture of viable bacteria in cecal contents (left) and feces (right) was performed. Values and error bars represent the average and 95% confidence intervals, respectively. IM: indomethacin; Strep: streptomycin

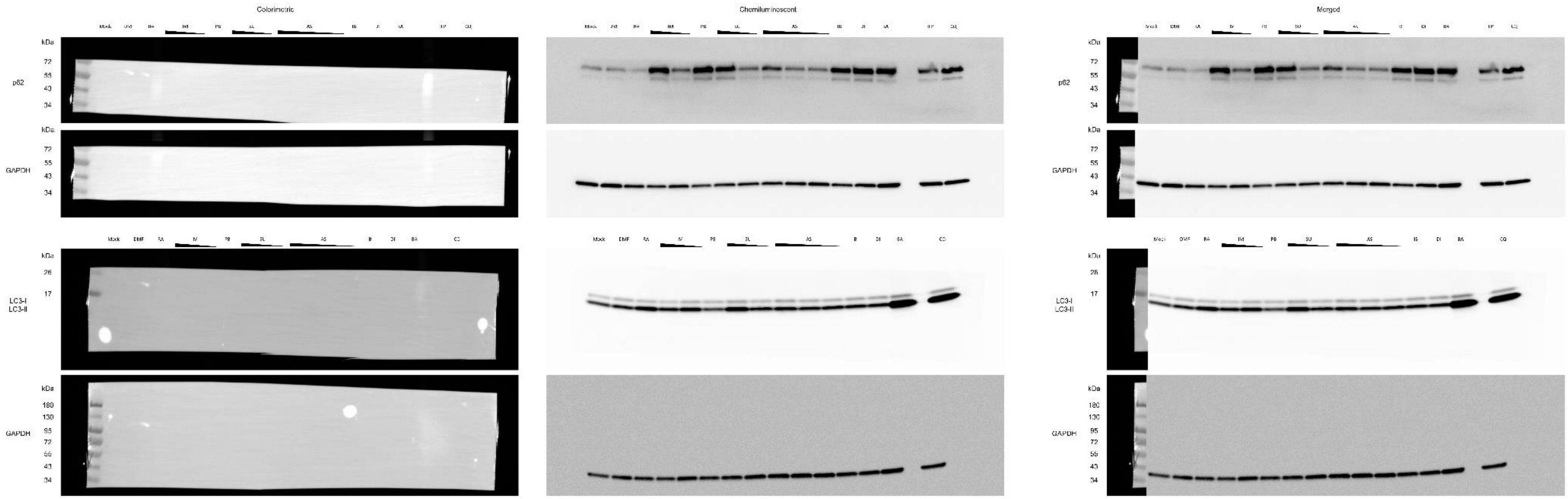
Colorimetric
Chemiluminescent
Merged
kDa
p62
GAPDH
LC3-I
LC3-II
Mock
DMF
CQ

69

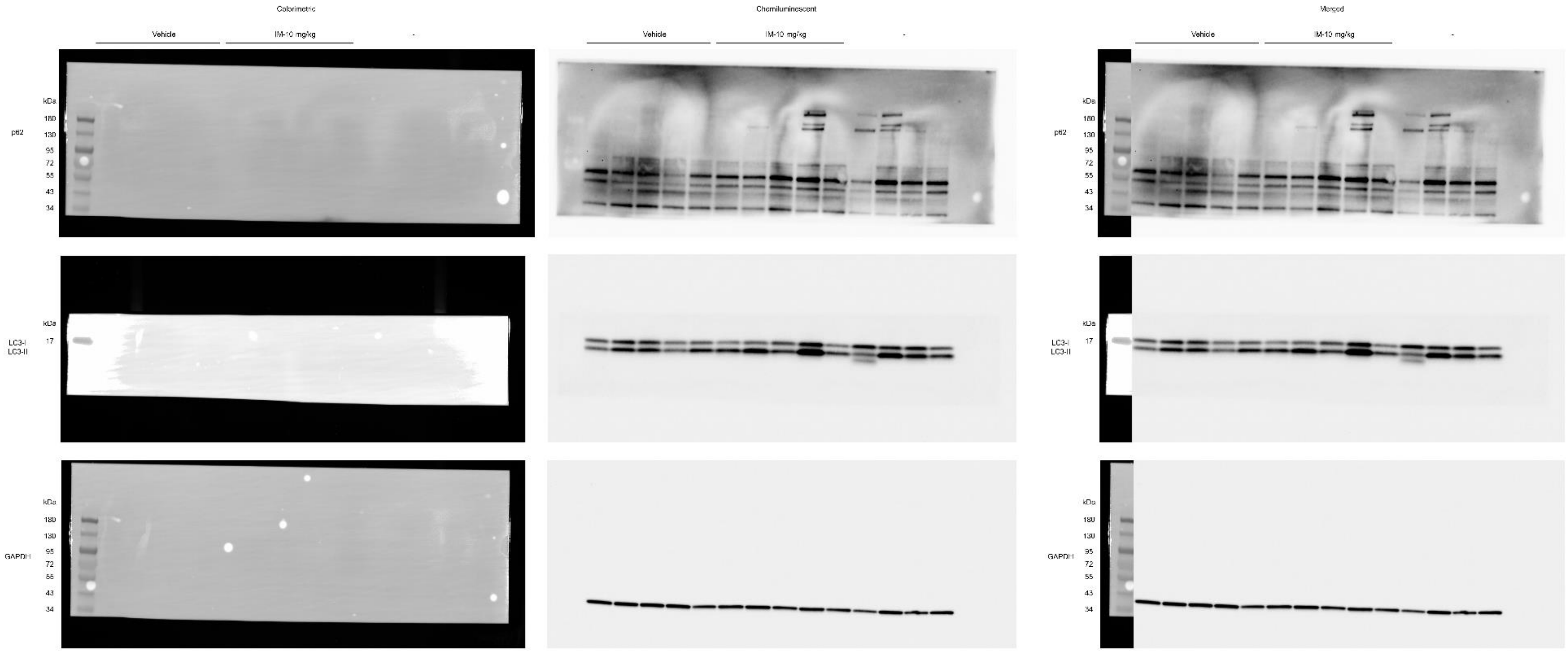
Colorimetric
Chemiluminescent
Merged
Vehicle
IM-10 mg/kg
-
kDa
180
130
95
72
55
43
34
p62
17
LC3-I
LC3-II
GAPDH

70
